# Identification of a novel RMST-ALK rearrangement in advanced lung adenocarcinoma and durable response to ceritinib: A case report

**DOI:** 10.3389/fonc.2022.913838

**Published:** 2022-08-01

**Authors:** Hui Li, Yixiao Deng, Bin Chen, Yajie Xiao, Jie Yang, Qionghui Liu, Gengpeng Lin

**Affiliations:** ^1^ Department of Pathology, The First Affiliated Hospital of Sun Yat-sen University, Guangzhou, China; ^2^ The Genetic Analysis Department, YuceBio Technology Co., Ltd., Shenzhen, China; ^3^ Department of Interventional Radiology, The First Affiliated Hospital of Sun Yat-sen University, Guangzhou, China; ^4^ Department of Pulmonary and Critical Care Medicine, The First Affiliated Hospital of Sun Yat-sen University, Institute of Pulmonary Diseases, Sun Yat-sen University, Guangzhou, China

**Keywords:** *RMST-ALK* rearrangement, non-small cell lung cancer, ceritinib, next-generation sequencing, sensitive

## Abstract

Next-generation sequencing technology has enabled the identification of fusion partners of anaplastic lymphoma kinase (*ALK*) in non-small cell lung cancer, and various *ALK* fusion partners have been confirmed. Here, a novel rhabdomyosarcoma 2-associated transcript (*RMST*)-*ALK* rearrangement was identified in an 80-year-old Chinese man with advanced lung adenocarcinoma. The patient was prescribed ceritinib and achieved a partial response, which has been sustained for more than 18 months. This is the first report of the *RMST-ALK* rearrangement, and we showed that a patient with lung adenocarcinoma carrying this rearrangement can benefit from ceritinib treatment; therefore, this is a significant finding in clinical practice.

## Introduction

Lung cancer is currently the third most common cancer worldwide (12.3% of all cancer diagnoses) and the leading cause of cancer-related mortality (21.4% of all cancer deaths) (1). Non-small cell lung cancer (NSCLC) accounts for approximately 85% of primary lung tumors (2). Molecular typing is the premise for the implementation of targeted therapy in NSCLC. Commonly mutated genes in lung adenocarcinoma in Eastern populations include *EGFR* (~60%), *KRAS* (~11%), and anaplastic lymphoma kinase (*ALK*; ~2%), and mutations have also been identified in *MET* (1%), *HER2* (~2%), *BRAF* (~2%), *RET* (1%–4%), and *ROS1* (~2%) (3, 4).

In most *ALK* rearrangements, one breakpoint of *ALK* often occurs at intron 19, which results in dissociation of the 3′ end of exons 20–29 from 5′ end sequences. The other breakpoint affects a diverse group of genes that contribute to the fusion oncogene, including a different gene promoter and a series of 5′ exons of variable lengths and properties, which predominantly share the ability to self-associate (5). Numerous fusion oncoproteins have been identified in various tumor types, of which echinoderm microtubule-binding-like protein 4 (*EML4*)*-ALK* is the primary fusion product (6). The ASCEND-4 and ASCEND-8 trials determined that first-line ceritinib treatment was efficacious in patients with advanced *ALK* fusion-positive NSCLC (7, 8). Here, we present our experience with a patient with lung adenocarcinoma with a rare *ALK* rearrangement who had a remarkable response after ceritinib treatment.

## Case presentation

**Figure 1:**
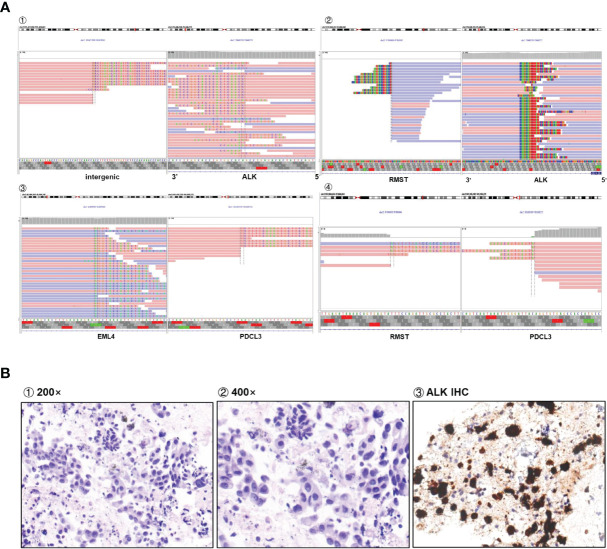
**(A)** Sequencing reads of multiple rearrangements are shown by the Integrative Genomics Viewer. **(B)** Positive ALK expression detected by IHC assay.

**Figure 2:**
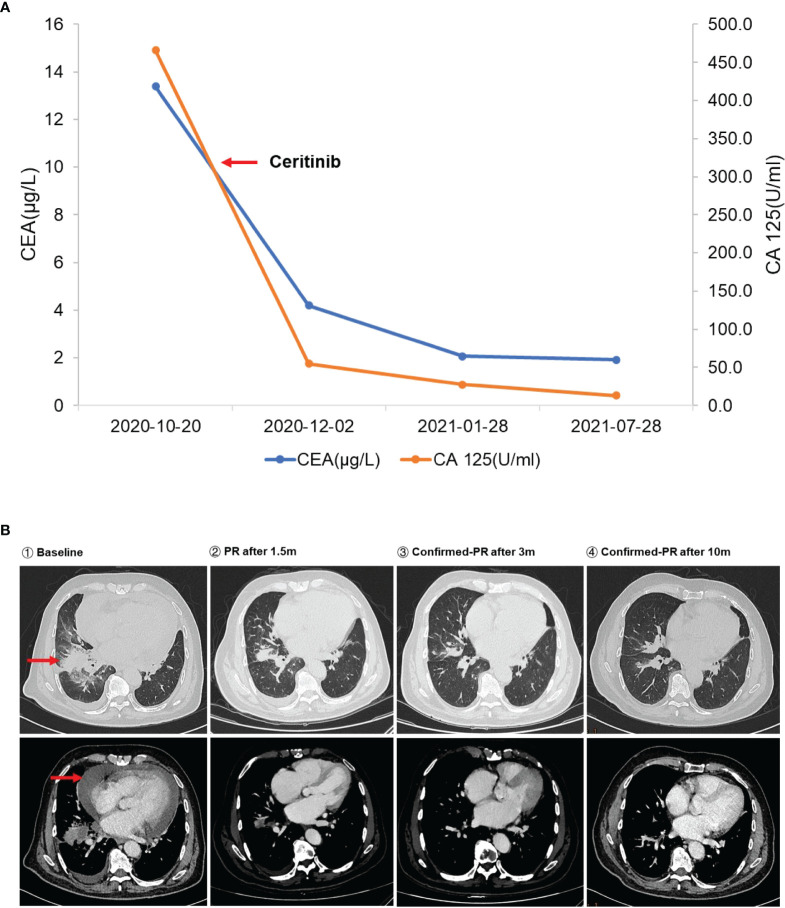
**(A)** The changes in serum carcinoembryonic antigen (CEA) and CA 125 level during ceritinib treatment. The left red arrow means initiation of ceritinib. **(B)** Dynamic imaging of lung lesions at different stages of treatment. The red arrows indicate the baseline lesion and pericardial effusion.

An 80-year-old Chinese man who had a more than 50-year smoking history and occasionally consumed alcohol was admitted to the local hospital in October 2020 because of hoarseness, shortness of breath after activity, decreased activity endurance, and chest tightness. The patient was otherwise in good health and did not have a history of hypertension, diabetes, coronary heart disease, pneumonia, tuberculosis, or other infectious disease. In 2007, he had undergone cystectomy for bladder cancer, and a percutaneous urine bag was indwelled for a long period after the operation. The patient underwent a physical examination and a series of auxiliary examinations. Enhanced computed tomography (CT) showed a mass in the middle and lower lobes of the right lung (57 × 35 mm), which was considered central lung cancer with obstructive inflammation and segmental atelectasis, and pericardial metastasis. The pathological report of the puncture effusion smear of the lung tumor under the fiberoptic bronchoscope indicated adenocarcinoma. Therefore, the patient was diagnosed with stage IVA non-small cell lung cancer (T3N2M1), ECOG PS 2. Next-generation sequencing (NGS) targeting 1,267 genes identified a rare novel rhabdomyosarcoma 2-associated transcript (*RMST*)-*ALK* translocation (R5′UTR: A20) and an *ALK*-intergenic (A19: intergenic) rearrangement (**Figure 1A**). The immunohistochemical (IHC) results confirmed that the tumor was *ALK* fusion positive (**Figure 1B**). Besides, no *EGFR* mutations and *ROS1* rearrangements were found. Based on the genetic test results, the patient was administered ceritinib treatment (450 mg P.0 QD) in November 2020. After treatment, his CEA declined from 13.38 to 4.2 μg/l, and his CA 125 declined from 465.7 to 54.6 U/ml (**Figure 2A**). Chest CT demonstrated significantly reduced hydropericardium 1.5 months later, confirming a partial response (**Figure 2B**). As of July 2022, the patient has retained a partial response to ceritinib treatment.

## Discussion

To our knowledge, this is the first case of an *RMST-ALK* rearrangement based on the fusion of the 5′ UTR of *RMST* and exon 20 of *ALK*. *RMST*, a long non-coding RNA, regulates mRNA and protein expression in the cytoplasm and is expressed in cervical cancer and triple-negative breast cancer (9). The *RMST-ALK* fusion is not currently documented in the COSMIC fusion database (https://cancer.sanger.ac.uk/cosmic/fusion) or the Quiver fusion database (http://quiver.archerdx.com/). The IVG view suggests that the *RMST-ALK* rearrangement may be transcribed in the opposite direction (“antisense rearrangement”). “Non-functional rearrangements” involving the *ALK* locus have been described, in which the reading frame of one or both genes is disrupted, *ALK* fuses with non-coding intergenic DNA, or genes are transcribed in the opposite orientation. These “non-functional” rearrangements might indicate *ALK* fusions by fluorescence *in situ* hybridization (FISH) (10). There are also some guidelines and expert consensus that DNA-seq NGS may identify atypical or intergenic fusions (11). Another case of an antisense rearrangement of *ALK* was previously reported, in which a complex tripartite rearrangement involving multiple DNA fusion junctions was formed between *YY1P2* downstream, *EML4*, and *ALK*. This fusion was detected by both FISH and IHC. The authors speculated that a complex structural mutation may have occurred in this region, ultimately producing a codable RNA sequence that generates a functional fusion transcript (12). Through bioinformatics mining and analysis of the raw data, we found that the sample in our case also had other genetic rearrangements: *EML4* and *RMST* fragments were fused to *PDCL3* (**Figure 1A**). This was validated by D5F3 IHC (**Figure 1B**), indicating that this *ALK* rearrangement could activate the self-phosphorylation of *ALK* and trigger its downstream signaling pathways, although the exact mechanism remains to be determined.

The gold standard for detecting *ALK* rearrangements is FISH or IHC, but neither method can identify a specific fusion form. Therefore, the detection of *ALK* rearrangements by targeted next-generation sequencing, such as DNA-based and RNA-based NGS, may be a good complementary approach to precisely identify rare or novel *ALK* fusion variants to guide targeted therapy with *ALK* inhibitors in patients with NSCLC (13–15). Crizotinib, which was approved by the US Food and Drug Administration (FDA) in 2011, was the first ALK-TKI approved for patients with ALK-positive NSCLC. The second-generation *ALK* TKI alectinib obtained expedited approval as first-line medication based on the results of the ALEX trial. Ceritinib is also a second-generation *ALK* TKI and is 20 times as potent as crizotinib against *ALK-*positive NSCLC, has significant antitumor activity against both crizotinib-sensitive and crizotinib-resistant tumors, and has better efficacy in the Asian population (16). Based on the results of ASCEND-4 and ASCEND-8 studies, the FDA and China National Medical Products Administration approved ceritinib as first-line treatment for patients with advanced NSCLC with *ALK* fusions in May 2017 and May 2020, respectively. Exploratory analysis of the ASCEND-1 study found that ceritinib was effective in *ALK*-rearranged NSCLC, including common *EML4-ALK* V3 and V1 variants, and novel *ALK* rearrangements, such as *CRIM1-ALK* and *CLTC-ALK*. Ceritinib is active against almost all *ALK*-resistant mutations found in patients pretreated with ALK inhibitors (17). Furthermore, ceritinib was found to be more effective and safe and to comply with treatment when administered with meals at 450 mg. Compared to patients receiving crizotinib, patients receiving ceritinib or alectinib showed better efficacy and had significantly longer progression-free survival (8, 18). Because ceritinib was more economically eligible than alectinib, the patient chose ceritinib for treatment. After 1.5 months of treatment, the CT scan showed that the right lung mass was slightly smaller than before, confirming a partial response. During more than 18 months of follow-up, the patient had no gastrointestinal adverse reactions such as diarrhea, nausea, vomiting, or abdominal pain and was in good living condition. Since *RMST-ALK* is a novel rearrangement, the mechanism of activation of *RMST* for this novel *ALK* rearrangement remains unclear, as dose has its ability to confer drug resistance. Further functional studies and clinical follow-up are required.

In conclusion, we report a novel case of *RMST-ALK* rearrangement in NSCLC with a durable response to ceritinib. This case may provide valuable information on the reaction to ceritinib of NSCLC patients with *RMST-ALK* rearrangement.

## Data availability statement

The datasets presented in this article are not readily available because of ethical and privacy restrictions. Requests to access the datasets should be directed to the corresponding author/s.

## Ethics statement

Written informed consent was obtained from the individual(s) for the publication of any potentially identifiable images or data included in this article.

## Author contributions

GL and QL: supervision, funding acquisition. HL, YD, and BC: conceptualization, methodology, writing—original draft preparation, writing—review and editing. YX and JY: formal analysis. All authors contributed to the article and approved the submitted version.

## Funding

This work was supported by the Guangdong Finance Foundation for Industrial Technology Research and Development [grant number 20160907].

## Conflict of interest

Authors YD, YX and JY are employed by YuceBio Technology Co., Ltd.

The remaining authors declare that the research was conducted in the absence of any commercial or financial relationships that could be construed as a potential conflict of interest.

## Publisher’s note

All claims expressed in this article are solely those of the authors and do not necessarily represent those of their affiliated organizations, or those of the publisher, the editors and the reviewers. Any product that may be evaluated in this article, or claim that may be made by its manufacturer, is not guaranteed or endorsed by the publisher.

## References

[1] Siegel RL Miller KD Fuchs HE Jemal A. Cancer statistics, 2022. CA Cancer J Clin (2022) 72(1):7–33. doi: 10.3322/caac.21708 35020204

[2] Gan J Fang W Zhang L. Therapy of lung cancer in China: introducing the special collection. Ther Adv Med Oncol (2021) 13:17588359211038199. doi: 10.1177/17588359211038199 34413904 PMC8369857

[3] Xue X Asuquo I Hong L Gao J Dong Z Pang L. Catalog of lung cancer gene mutations among Chinese patients. Front Oncol (2020) 10:1251. doi: 10.3389/fonc.2020.01251 32850378 PMC7417348

[4] Zhang Q Wu C Ding W Zhang Z Qiu X Mu D. Prevalence of ROS1 fusion in Chinese patients with non-small cell lung cancer. Thorac Cancer (2019) 10(1):47–53. doi: 10.1111/1759-7714.12899 30468296 PMC6312842

[5] Mariño-Enríquez A Dal Cin P. ALK as a paradigm of oncogenic promiscuity: different mechanisms of activation and different fusion partners drive tumors of different lineages. Cancer Genet (2013) 206(11):357–73. doi: 10.1016/j.cancergen.2013.07.001 24091028

[6] Shaw AT Engelman JA. ALK in lung cancer: past, present, and future. J Clin Oncol (2013) 31(8):1105–11. doi: 10.1200/JCO.2012.44.5353 PMC4209068 23401436

[7] Soria JC Tan DSW Chiari R Wu YL Paz-Ares L Wolf J. First-line ceritinib versus platinum-based chemotherapy in advanced ALK-rearranged non-small-cell lung cancer (ASCEND-4): a randomised, open-label, phase 3 study. Lancet (2017) 389(10072):917–29. doi: 10.1016/S0140-6736(17)30123-X 28126333

[8] Cho BC Kim DW Bearz A Laurie SA McKeage M Borra G. ASCEND-8: A randomized phase 1 study of ceritinib, 450 mg or 600 mg, taken with a low-fat meal versus 750 mg in fasted state in patients with anaplastic lymphoma kinase (ALK)-rearranged metastatic non-small cell lung cancer (NSCLC). J Thorac Oncol (2017) 12(9):1357–67. doi: 10.1016/j.jtho.2017.07.005 28729021

[9] Wang L Liu D Wu X Zeng Y Li L Hou Y. Long non-coding RNA(LncRNA) RMST in triple-negative breast cancer (TNBC): Expression analysis and biological roles research. J Cell Physiol (2018) 233(10):6603–12. doi: 10.1002/jcp.26311 29215701

[10] Rosenbaum JN Bloom R Forys JT Hiken J Armstrong JR Branson J. Genomic heterogeneity of ALK fusion breakpoints in non-small-cell lung cancer. Mod Pathol (2018) 31(5):791–808. doi: 10.1038/modpathol.2017.181 29327716

[11] Li W Zhang J Wang Z Li L Ma J Zhou X. Guidelines for clinical practice of ALK fusion detection in non-small-cell lung cancer: a proposal from the Chinese RATICAL study group. J Natl Cancer Center (2021) 1(4):123–31. doi: 10.1016/j.jncc.2021.07.005 PMC11256616 39036803

[12] Li W Liu Y Li W Chen L Ying J. Intergenic breakpoints identified by DNA sequencing confound targetable kinase fusion detection in NSCLC. J Thorac Oncol (2020) 15(7):1223–31. doi: 10.1016/j.jtho.2020.02.023 32151779

[13] Qiu H Li Q Xiao Y Wu D Meng R. A novel intergenic region between KLHL31 and LRRC1-ALK exon 20 fusion variant in advanced lung adenocarcinoma and its remarkable response to ALK inhibitor. J Thorac Oncol (2021) 16(4):e21–3. doi: 10.1016/j.jtho.2020.12.016 33781445

[14] Hou X Xu H Chen L. SRBD1-ALK, a novel ALK fusion gene identified in an adenocarcinoma patient by next-generation sequencing. J Thorac Oncol (2019) 14(4):e72–3. doi: 10.1016/j.jtho.2018.11.027 30922581

[15] Chen HF Wang WX Xu CW Huang LC Li XF Lan G. A novel SOS1-ALK fusion variant in a patient with metastatic lung adenocarcinoma and a remarkable response to crizotinib. Lung Cancer (2020) 142:59–62. doi: 10.1016/j.lungcan.2020.02.012 32114282

[16] Shaw AT Kim DW Mehra R Tan DS Felip E Chow LQ. Ceritinib in ALK-rearranged non-small-cell lung cancer. N Engl J Med (2014) 370(13):1189–97. doi: 10.1056/NEJMoa1311107 PMC4079055 24670165

[17] Tan DS Thomas M Kim DW Szpakowski S Urban P Mehra R. Genetic landscape of patients with ALK-rearranged non-small-cell lung cancer (NSCLC) and response to ceritinib in ASCEND-1 study. Lung Cancer (2022) 163:7–13. doi: 10.1016/j.lungcan.2021.11.007 34890832

[18] Peters S Camidge DR Shaw AT Gadgeel S Ahn JS Kim DW. Alectinib versus crizotinib in untreated ALK-positive non-Small-Cell lung cancer. N Engl J Med (2017) 377(9):829–38. doi: 10.1056/NEJMoa1704795 28586279

